# Using TAS Test online hand‐tapping tests to detect cognitive decline in a large community cohort of older Australians

**DOI:** 10.1002/alz.091929

**Published:** 2025-01-09

**Authors:** Jane E Alty, Xinyi Wang, Aidan Bindoff, Rebecca J St George, Eddy Roccati, Larissa Bartlett, Katherine Lawler, Alastair J Noyce, Son Tran, Anna E King, Quan Bai, James C Vickers

**Affiliations:** ^1^ Royal Hobart Hospital, Hobart, TAS Australia; ^2^ School of Medicine, University of Tasmania, Hobart, TAS Australia; ^3^ Wicking Dementia Research and Education Centre, University of Tasmania, Hobart, TAS Australia; ^4^ School of Psychological Sciences, University of Tasmania, Hobart, TAS Australia; ^5^ La Trobe University, Melbourne, VIC Australia; ^6^ Queen Mary University of London, London, England UK; ^7^ School of Information and Communication Technology, University of Tasmania, Hobart, TAS Australia

## Abstract

**Background:**

Finding low‐cost, accessible methods to detect people with early‐stage Alzheimer’s disease is a research priority for neuroprotective drug development. Subtle motor impairment of gait occurs years before episodic memory decline but there has been little investigation of whether self‐administered hand motor tests can detect this pre‐symptomatic period. This study evaluated how home‐based unsupervised keyboard tapping tests from the TAS Test protocol predict episodic memory performance in a sample of older adults without overt cognitive impairment, as a potential indicative measure of early Alzheimer’s disease.

**Method:**

1,140 community participants (65.7 ± 7.4 years old; 73% female) without cognitive impairment from the ISLAND cohort study completed a 40‐second single key tapping test, a 60‐second alternate key tapping test, and a 40‐second sequence tapping test from TAS Test. Participants also completed validated CANTAB cognitive tests of episodic memory, working memory and executive function. Frequency, variability, key press duration and accuracy scores were calculated for each tapping test. Generalized linear models examined associations between keyboard tapping and cognitive performance, adjusted for confounders including age, sex, depression, anxiety and education.

**Result:**

Combination of motor features of the single key (R^2^_adj_ = 8.0%, ΔAIC = 3.7), alternate key (R^2^_adj_ = 7.9%, ΔAIC = 2.8), and sequence tapping tests (R^2^_adj_ = 8.2%, ΔAIC = 8.4) improved estimation of episodic memory performance relative to models with demographic and mood confounders only (R^2^_adj_ = 7.3%). Only tapping features of tests involving sequence tapping (R^2^_adj_ = 6.3%, ΔAIC = 2.5) improved estimation of working memory. Tapping features of single key tapping tests (R^2^_adj_ = 15.8%, ΔAIC = 8.3) and sequence tapping tests (R^2^_adj_ =16.5%, ΔAIC = 13.7) improved the estimation of executive function performance.

**Conclusion:**

Brief unsupervised and self‐administered online keyboard tapping tests predict asymptomatic episodic memory decline in a sample of older adults. This provides a potential low‐cost and brief home‐based method for risk stratification of enriched cohorts for further assessment.

